# GABARAPL1 Promotes AR+ Prostate Cancer Growth by Increasing FL-AR/AR-V Transcription Activity and Nuclear Translocation

**DOI:** 10.3389/fonc.2019.01254

**Published:** 2019-11-15

**Authors:** Bing Su, Lijuan Zhang, Shenglin Liu, Xiaofan Chen, Wei Zhang

**Affiliations:** ^1^Xinxiang Key Lab of Translational Cancer Research, The Third Affiliated Hospital, Xinxiang Medical University, Xinxiang, China; ^2^Biomedical Research Institute, Shenzhen Peking University - The Hong Kong University of Science and Technology Medical Center, Shenzhen, China

**Keywords:** prostate cancer, androgen receptor, AR variant, GABARAPL1, androgen deprivation therapy

## Abstract

The next generation Androgen receptor (AR)-targeted therapies are now in widespread clinical use and prolong prostate cancer (CaP) patient survival. However, the therapies are not curative due to diverse range of resistance mechanisms. AR variants (AR-V), one major mechanism of resistance, has recently gained momentum. Here, we found that GABARAPL1 knockdown inhibits the growth of AR-positive LNCaP and CWR22rv1 CaP cells *in vitro* and *in vivo*, decreases AR/AR-V transcription activity and AR nuclear translocation. Pulldown assay shows that both of Full-length (FL)-AR and AR-V were able to interact with GABARAPL1, suggesting that GABARAPL1 may play its role through directly scaffolding AR. The further analysis from Oncomine database showed that negative correlation between GABARAPL1 expression and 5-years survival in CaP cases. Our findings have identified GABARAPL1 as critical regulator of FL-AR/AR-V, suggesting the potential benefit of targeting GABARAPL1 to treat AR-positive CaP that is resistant to next generation AR inhibitors.

## Introduction

Prostate cancer (CaP) is the most common cancer in men and the second leading cause of male cancer mortality in the United States (1). Despite initial robust responses to first-line androgen deprivation therapy (ADT), nearly all patients with advanced disease inevitably progress to a lethal castration-resistant prostate cancer (CRPC). An abundance of data demonstrated that androgen receptor (AR) signaling remains active and continues to drive the growth of CRPC following ADT. This notion is further supported by the success of FDA-approved second generation AR-targeting reagents abiraterone acetate (2) and enzalutamide (3), which have been shown to extend overall survival of patients with CRPC (4). While effective, therapies targeting AR are not curative, and CRPC patients will eventually develop secondary resistance (5, 6).

The production of c-terminally truncated AR variant (AR-V) proteins lacking the AR ligand binding domain (LBD) has been proposed to be a major mechanism driving resistance to conventional and next-generation ADT (7). These ligand-independent AR-Vs are constitutively active and insensitive to anti-androgens or androgen ablation therapy (8). To overcome this resistance mechanism, it is essential to develop novel strategies to simultaneously disrupt the full-length AR (FL-AR) and AR-V.

The GABARAPL1 (known as GEC1 or ATG8L) was initially identified as an early estrogen-induced gene in quiescent guinea-pig endometrial glandular epithelial cells (GEC). Reduced GABARAPL1 expression was observed in various cancer cell lines (9). A study of a cohort of 265 breast adenocarcinoma biopsies demonstrated that patients with high GABARAPL1 mRNA levels had a lower risk of recurrence (10).

We previously demonstrated that GABARAPL1 mediates androgen-regulated autophagy in CaP cells (11). In current study, we show that knockdown of GABARAPL1 inhibits CaP cells growth *in vitro* and *in vivo*, decreases the transcriptional activity of FL-AR/AR-V and their nuclear translocation through directly scaffolding, suggesting that GABARAPL1 is a critical regulator of FL-AR/AR-V. Our findings indicate that GABARAPL1 may be a potential therapeutic target in FL-AR/AR-V-positive CRPC that is resistant to the next generation AR inhibitors.

## Materials and Methods

### Antibodies and Reagents

The following primary antibodies (Ab) were used: rabbit polyclonal for AR, tubulin and GST (Cell Signaling Technology, Beverly, MA), GABARAPL1 (Proteintech, Rosemont, IL). Mouse monoclonal for AR (Abcam, Shanghai, China), β-Actin (Santa Cruz Biotechnology, Santa Cruz, CA), Myc (Applied Biological Materials, Richmond, BC, Canada). CellTiter 96® AQueous MTS Reagent Powder was obtained from Promega (Madison, WI), charcoal-stripped fetal bovine serum (CS-FBS) was from Gibco (Grand Island, NY), BD MartigelTM Matrix (BD Biosciences, San Jose, CA) and p3XFLAG-Myc-CMV™-24 Expression Vector (Sigma-Aldrich, St. Louis, MO).

### Cell Culture

LNCaP, CWR22Rv1, PC-3, DU145, and HEK293T cells were purchased from the Chinese Academy of Cell Bank. LNCaP and CWR22Rv1 cells were cultured in RPMI1640 media supplemented with 10% FBS and incubated at 37°C in a humidified incubator containing 5% CO_2_. PC-3 cells were cultured in DMEM/F12 media supplemented with 10% FBS. DU145 cells were cultured in DMEM media supplemented with 10% FBS. For androgen-addition culture medium, CS-FBS was used as a substitute for FBS, and cells were cultured overnight prior to the addition of 10 nM Dihydrotestosterone (DHT).

### Plasmids and Constructs

GST-GABARAPL1 fusion plasmid was made by PCR amplification and then confirmed by sequencing. Forward and revere primers contain an EcoR I and Sal I site to facilitate cloning into the pGEX-5X vectors. The p3XFLAG-Myc-CMV™-24-AR-NTD or DBD truncated construct was produced by PCR amplification with forward primer containing EcoRI site and reverse primer containing Sal I site. The p3XFLAG-Myc-CMV™-24-AR-LBD construct was produced by PCR amplification with forward primer containing Not I site and reverse primer containing Sal I site. The primers were listed in Supplementary Table 1. Human AKT1 primer sequences are from Guo's study (12).

### Transfection

Lentiviruses expressing pLKO.1-shRNA-GABARAPL1 (shGab1) or control vectors were generated by transfection into HEK293A cells as previously published (11). Prostate cancer cells were transduced with conditioned medium containing lentivirus particles and selected by puromycin. The knockdown efficiency was evaluated by real-time RT-PCR analysis. HEK293T cells were transiently transfected with p3XFLAG-Myc-CMV™-24-truncated AR plasmid and cell lysates were collected after 48 h.

### Cell Growth Assay

The growth of prostate cancer cells infected with shRNA-GABARAPL1 or control shRNA were evaluated using MTS assay following the manufacturer's protocol.

### Western Blot Analysis

Cell lysates were generated and Western blotting with AR, GABARAPL1, GST, Myc, and βactin antibodies was carried out as described previously (13).

### Xenograft Prostate Cancer Model in Nude Mice

Male BALB/c nude mice (4–6 weeks) were purchased from Beijing HFK Bioscience Company (Beijing, China) and maintained under specific pathogen-free (SPF) condition. CWR22rv1 cells (106) with stably infection of shRNA-GABARAPL1 or control shRNA were suspended in 50 μL RPMI1640 and 50 μL Martigel were randomly injected s.c. into the left and right flank of mice (5 mice/group). Tumor volume (cubic millimeters) was measured weekly using a caliper, applying the formula [volume = 0.52 × (width)2 × (length)] for approximating the volume of a spheroid. When the experiment ends, mice were terminally narcotized and sacrificed. This study was performed in accordance with animal use protocols approved by the Committee for the Ethics of Animal Experiments, Shenzhen Peking University-The Hong Kong University of Science and Technology Medical Center (SPHMC) (protocol number 2011-004). All animals were handled in accordance with the guidelines of the Committee for the Ethics of Animal Experiments, SPHMC.

### Luciferase Reporter Assay

LNCaP or CWR22rv1 cells harboring shRNA-GABARAPL1 were transiently transfected with AR-responsive pMMTV-luc reporter plasmid, together with pRL-CMV plasmid to normalize the transfection efficiency. The relative luciferase activities were calculated by normalizing the firefly luciferase activity to Renilla luciferase activity.

### Quantitative Reverse Transcriptase PCR (qRT-PCR)

Total RNA was isolated using TRIZOL Reagent (Sigma-Aldrich, St. Louis, MO) and reverse transcripted into cDNA using RevertAid First Strand cDNA Synthesis Kit (Thermo Scientific, Waltham, MA). Real-time PCR was performed using SYBR premix EX Taq (TaKaRa Bio, Dalian, China) and analyzed with CFX96 Real-Time System (BIO-RAD). Real-time primer sequences were listed in Supplementary Table 1. GAPDH was used as a housekeeping gene for the qRT-PCR reactions. Each test was done in triple replication and the 2–ΔΔCt method was used to calculate the expression of genes.

### Pulldown Assay

Lysates containing 500 μg of protein from LNCaP or CWR22rv1 cells were incubated with glutathione sepharose 4B beads bound to GST or GST-GABARAPL1 fusion proteins. After washing with RIPA buffer, the beads were subjected to immunoblot analysis probed with anti-AR antibody. Lysates of HEK293T cells transiently transfected with p3XFLAG-Myc-CMV™-24-truncated AR plasmids were incubated with glutathione sepharose 4B beads bound to GST or GST-GABARAPL1 fusion proteins. After washing with RIPA lysis buffer, the beads were subjected to immunoblot analysis probed with Myc antibody.

### Immunofluorescence Analysis

LNCaP cells were plated on glass cover-slips in 6-well plates in medium containing 10% CS-FBS overnight prior to the addition of 10 nM DHT for six additional hour. Cells were then fixed in 4% paraformaldehyde for 30 min and permeabilized by 0.1% Triton X-100 for 10 min. The cover slips were rinsed in PBS, blocked in 5% BSA for 2 h, and then incubated with mouse anti-AR antibody (1:500) and rabbit anti-tubulin (1:1,000). The cover slips were washed three times in PBS and incubated with CY3 anti-mouse secondary antibody (red) and Alexa Fluor488 (green) for 2 h. After three washes in PBS, cover-slips were mounted and cells visualized using a Zeiss LSM 510 fluorescence microscope with a ×60 objective.

### Statistical Analyses

Statistical significances between groups were determined by two-tailed student's *t*-test. All statistical analyses were performed using SPSS 16.0 software program. A *p* < 0.05 was considered significant.

## Results

### Knockdown of GABARAPL1 Inhibits the Growth of AR-Positive Prostate Cancer Cells *in vitro* and *in vivo*

**Figure 1:**
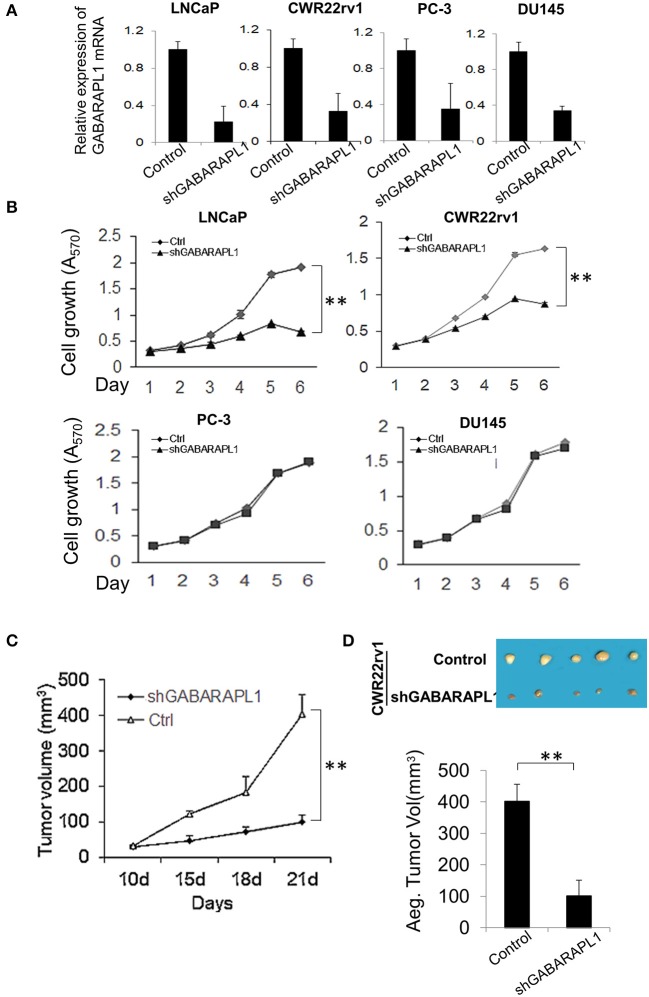
Downregulation of Gabarapl1 inhibits the growth of AR+ prostate cancer cells *in vitro* and *in vivo*. **(A)** CaP cells were transduced with sh-GABARAPL1 or sh-control lentivirus, then GABARAPL1 mRNA expression were validated using RT-qPCR. **(B)** AR-positive LNCaP and CWR22rv1 cells or AR-negative PC-3 and DU145 were transduced with sh-GABARAPL1 or sh-control lentivirus, and cell growth was evaluated. Ctrl: Control cells, shGABARAPL1: GABARAPL1 knockdown cells. Error bars, SE of three independent experiments; ^**^P < 0.01. **(C,D)** Tumor growth inhibition by knockdown of GABARAPL11 in mouse xenograft models of CWR22rv1 cells s.c. Representative pictures of tumor in nude mice (**D**, upper), and tumor size (**D**, lower). ^**^P < 0.01.

We previously demonstrated that overexpression of GABARAPL1 inhibits cell proliferation, invasion, and metastasis of CaP cells *in vitro* and *in vivo* (11, 13). Similar results were reported in breast cancer (10) and hepatocellular carcinoma (14), highlighting a role of GABARAPL1 as a tumor suppressor. However, the role of GABARAPL1 is controversial with a study showing that downregulation of GABARAPL1 suppresses tumorigenesis and metastasis in triple negative breast cancer cells (15). Hence, we further investigated the effect of GABARAPL1 on the growth of CaP cells using previously described shRNA-GABARAPL1 (11). Successful knockdown of GABARAPL1 mRNA expression was validated by RT-qPCR analysis (Figure 1A). Interestingly, knockdown of GABARAPL1 resulted in robust inhibition of growth of AR-positive LNCaP and CWR22rv1 cells (Figure 1B), but not AR-negative DU145 and PC-3 cells (Figure 1B), indicating that the inhibitory effect of GABARAPL1 knockdown on cell growth was likely caused by its effect on the AR. This observation is consistent with our previous finding that overexpression of GABARAPL1 inhibits the proliferation of LNCaP cells, but does not affect apoptosis (11).

To investigate whether GABARAPL1 knockdown affects primary tumorigenesis of AR-positive CaP *in vivo*, CWR22rv1 cells transduced with shGABARAPL1 were used in a xenograft CaP mouse model to evaluate tumorigenesis. Nude mice were injected subcutaneously with shGABARAPL1-CWR22rv1 cells, and tumor growth was monitored. As shown in Figure 1C, knockdown of GABARAPL1 decreased primary tumor growth. The volumes of the primary tumors generated from shGABARAPL1-CWR22rv1 cells were significantly lower than those from the control group (Figure 1D). These results indicate that inhibition of GABARAPL1 represses AR-positive CaP growth and tumorigenesis *in vitro* and *in vivo*.

### Knockdown of GABARAPL1 Decreases FL-AR/AR-V Transcriptional Activity and Nuclear Translocation Through Directly Scaffolding AR

**Figure 2:**
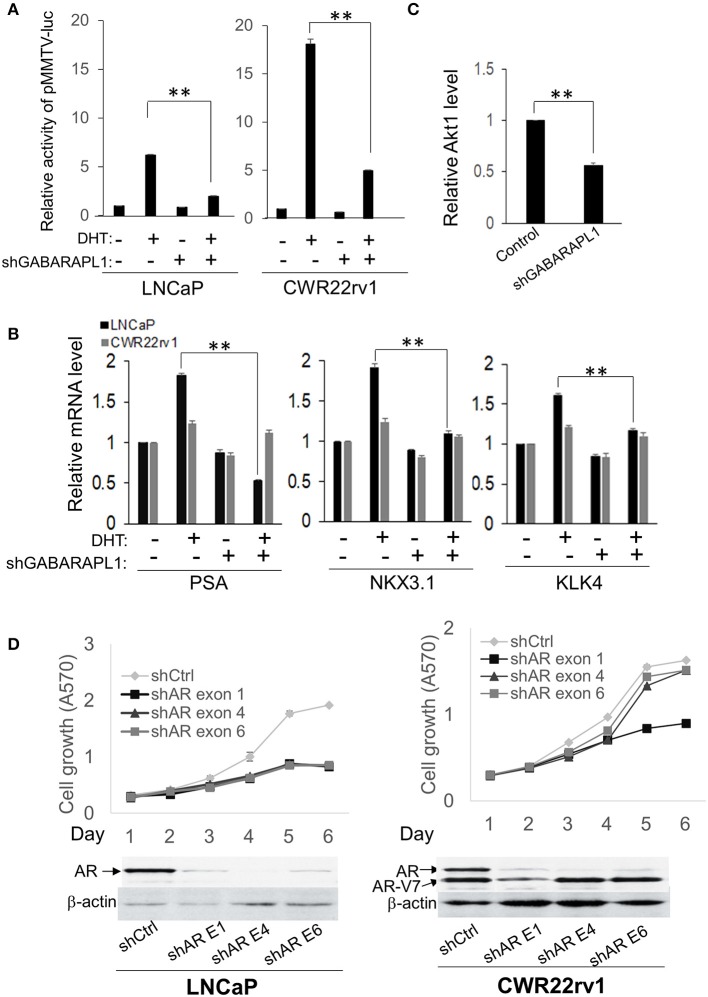
GABARAPL1 mediates FL-AR/AR-V transcriptional activity in LNCaP and CWR22rv1 cells. **(A)** LNCaP and CWR22rv1 cells were stably infected with shRNA-GABARAPL1 or control shRNA. These cells were tranfected with AR-responsive MMTV promoter reporter plasmids for 24 h and then treated with DHT (10 nM). MMTV promoter activities were presented as mean ± SE from three independent experiments. **(B)** Androgen-dependent AR target gene expression in LNCaP cells with knockdown of GABARAPL1. LNCaP cells were stably infected with shRNA-GABARAPL1 or control shRNA. These cells were treated with DHT (10 nM) for 24 h. mRNA expression of AR target genes (PSA, NKX3.1, and KLK4) was assessed by quantitative RT-PCR. **(C)** AR-V targeted AKT1 gene expression in CWR22rv1 cells with knockdown of GABARAPL1. **(D)**. CWR22rv1 cells were transfected and treated as in **(B)**. The growth was assessed at indicated time points (upper panel). The protein levels of AR or AR-V7 were assessed by Western blot analysis. β-actin was an internal control (lower panel).

Since our study showed that GABARAPL1 only affects cell growth in AR-positive CaP cells, and knockdown of GABARAPL1 does not alter AR level (Supplementary Figure 1), we postulate that GABARAPL1 may exert its activity through regulating the transcriptional activity of AR. AR-positive LNCaP and CWR22rv1 cells were stably transduced with shRNA-GABARAPL1 and transfected with AR-responsive MMTV-Luc reporter plasmids. AR reporter activity was inhibited by GABARAPL1 knockdown in both LNCaP and CWR22rv1 cells (Figure 2A). Expression of androgen-mediated activation of the AR target genes PSA, NKX3.1, and KLK4 were abolished in shRNA-GABARAPL1-infected LNCaP cells, but not significantly affected in CWR22rv1 cells (Figure 2B).

To further address whether GABARAPL1 is required for AR-V activity, androgen-independent AKT1 expression, which is uniquely transcriptionally activate alternative target for AR-V (12), was analyzed in CWR22rv1 cells. It was found that knockdown of GABARAPL1 decreases the expression of AKT1, indicating that downregulation of GABARAPL1 blocks AR-V activity in CWR22Rv1 cells (Figure 2C). These findings suggest that inhibition of growth of AR-positive CaP cells by knockdown of GABARAPL1 was through inhibiting the transcriptional activity of both FL-AR and the short form AR-V.

To confirm our hypothesis, we designed shRNA targeting exon 1, exon 4, or exon 6 of AR to examine whether FL-AR and/or AR-V contributes to the growth inhibitory effect of GABARAPL1 knockdown. Results showed that shRNA against exon 4 and 6 reduced the expression of FL-AR, while shRNA against exon 1 knocked down both FL-AR and AR-V7 (Figure 2D, lower left and right panel). As expected, all three shRNAs significantly reduced the growth of LNCaP cells (Figure 2D, upper left panel). However, only knockdown of FL-AR (shRNA against AR exon 4 or 6) did not affect CWR22Rv1 growth (Figure 2D, upper right panel). Conversely, ablation of both FL-AR and AR-V significantly inhibited growth of CWR22Rv1 cells to the same extent as knockdown of GABARAPL1 (Figure 2D, upper right panel). These findings strongly indicate that FL-AR and AR-V promotes cell proliferation in a redundant way, and GABARAPL1 ablation reduces cell growth by targeting both FL-AR and AR-V.

**Figure 3:**
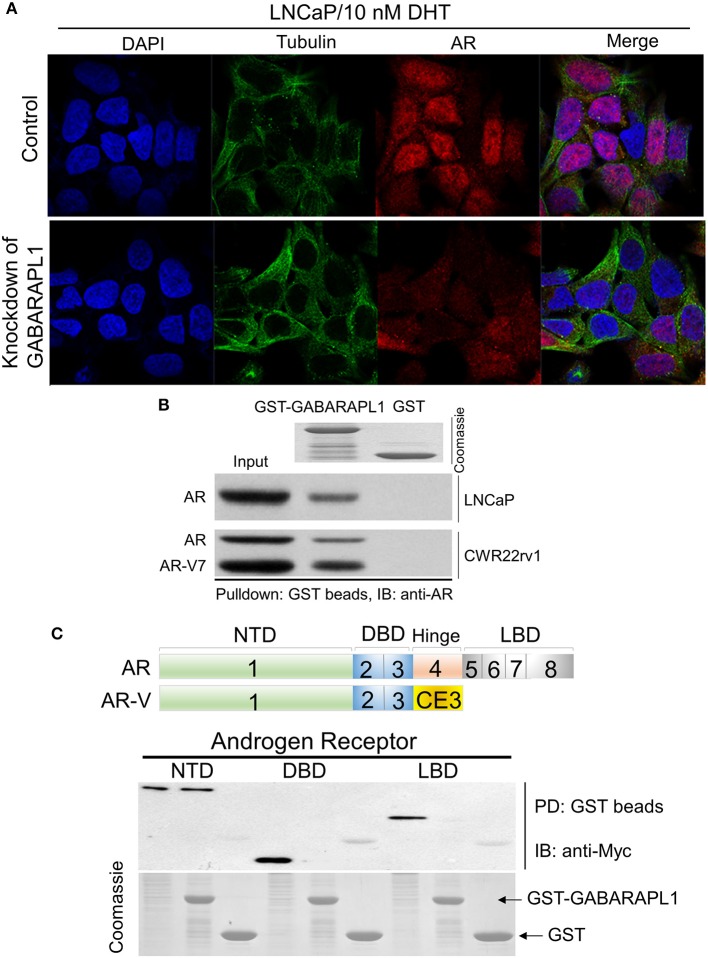
Knockdown of GABARAPL1 decreases AR nuclear translocation through directly scaffolding. **(A)** LNCaP cells were treated with DHT (10 nM) for 6 h to induce AR nuclear translocation. Cells were fixed and immunostained with antibodies against AR (red), and tubulin (green). **(B)** Beads containing GST or GST-GABARAPL1 fusion protein (Coomassie stained proteins in top panel) were incubated with LNCaP or CWR22rv1 lysates, washed and then blotted for bound FL-AR or AR-V protein. **(C)** Upper: schematic representation of AR and AR-V protein domains. Bottom: Glutathione-beads charged with GST-Gabarapl1 were used to pull down HEK293T/AR (NTD, DBD, and LBD) lysates, followed by detection of pull-down products using anti-Myc.

Several LC3/GABARAP family proteins have been implicated in receptor trafficking. GABARAPL1 was observed at the plasma membrane and could indicate a role for GABARAPL1 in membrane stabilization (16). Since GABARAPL1 functions in membrane fusion and is involved in receptor trafficking, we thought GABARAPL1 might affect FL-AR/AR-V transcriptional activity by interfering with AR nuclear translocation. Therefore, we investigated the effect of GABARAPL1-knockdown on AR nuclear translocation in LNCaP cells. AR was predominantly cytoplasmic in the absence of androgen in LNCaP cells. Following the treatment with DHT, AR largely translocated into the nucleus (Figure 3A). As expected, knockdown of GABARAPL1 by shRNA induced a marked reduction in AR nuclear accumulation (Figure 3A), suggesting that GABARAPL1 may decrease FL-AR/AR-V transcriptional activity through interference of AR nuclear translocation.

Substantial evidence shows that the LC3/GABARAP family proteins have functions unrelated to autophagy. For instance, GABARAP subfamily proteins serve as scaffolding proteins by recruiting ULK1 and beclin-1 to the nucleation site (16). GABARAPL1 has been shown to interact *in vitro* with two essential membrane receptors in the brain: gamma-aminobutyric acid, type A receptor (GABAAR) (17) and kappa opioid receptor (KOR) (18). GABARAPL1 contributes to the neuronal signal transmission by aiding in the transport of these membrane receptors to the cell surface (19). These findings suggest that GABARAPL1 might interfere with AR nuclear translocation by directly scaffolding AR. Hence, we examined the potential interaction between GABARAPL1 and AR using protein extracts from LNCaP or CWR22rv1 cells in a GST pull-down experiment. We observed that FL-AR was pulled-down with GST-GABARAPL1 in LNCaP cells, and FL-AR/AR-V were pulled-down in CWR22rv1 cells, indicating that both FL-AR and AR-V were able to interact with GABARAPL1 (Figure 3B).

The AR is structurally composed of an amino-terminal domain (NTD), a DNA-binding domain (DBD), and a carboxy-terminal ligand-binding domain (LBD) (Figure 3C, upper panel). To further identify the binding sites between GABARAPL1 and AR, we performed pulldown assays. GST-GABARAPL1 beads were incubated with cell lysates from HEK293T cells that were forced to overexpress truncated AR (AR-NTD, AT-DBD, and AR-LBD). Probing the pulldown products with anti-Myc revealed that AR-NTD region was associated with GABARAPL1 (Figure 3C, lower panel).

### The Negative Correlation Between GABARAPL1 Expression and 5-Year Survival Rate in Human CaP Tissues

**Figure 4:**
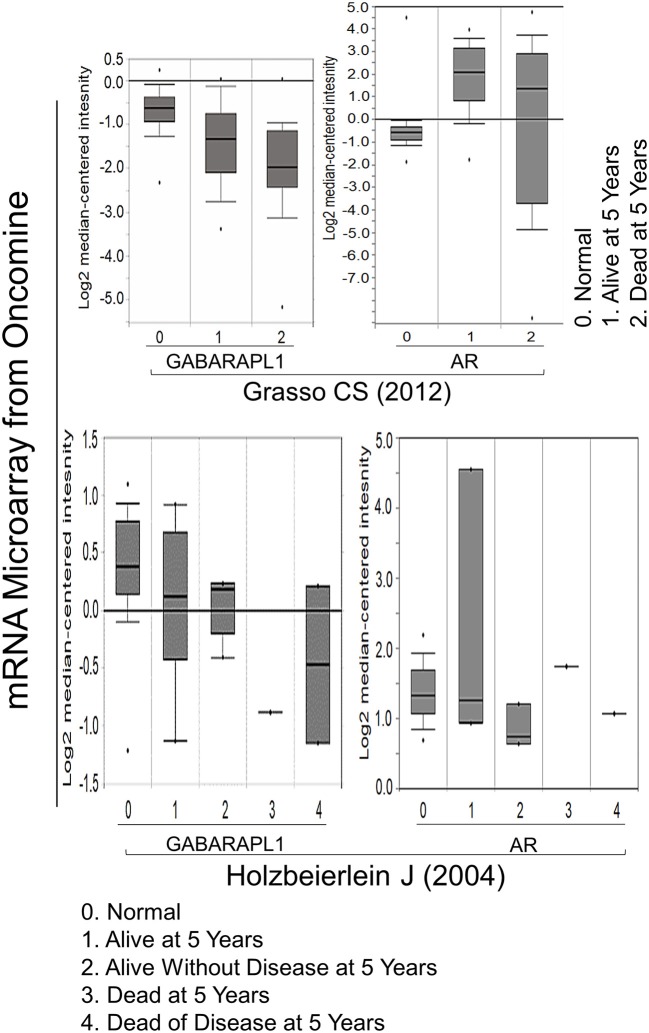
The negative correlation between GABARAPL1 expression and 5-year survival in CaP cases. The data were obtained from two studies available on the Oncomine website: Grasso et al. (20) and Holzbeierlein et al. (21).

Whether GABARAPL1 expression associates with cancer remains controversial. We and other researchers previously observed reduced GABARAPL1 expression in CaP, breast cancer (10) and hepatocellular carcinoma (14), suggesting that GABARAPL1 may serve as a tumor suppressor. However, our current findings indicate that knockdown of GABARAPL1 inhibits CaP cells growth. The analysis of Oncomine database validated reduced GABARAPL1 expression in CaP tissues compared with normal control as previously reported (13). Interestingly, low level of GABARAPL1 in CaP tissues correlates with a shorter survival rate, but there is no correlation between AR and 5-year survival rate (Figure 4), supporting GABARAPL1 as a chaperone protein for AR. There was no association between GABARAPL1 expression and 5-year survival rate of breast or colon cancer (Supplementary Figures 2, 3), suggesting that the putative function by GABARAPL1 would be tissue-type specific.

## Discussion

All of the current standard of care therapies for advanced CaP act through disrupting AR signaling by lowering androgen levels or preventing androgen-AR binding, which depends on an intact AR C-terminal LBD. Although these agents prolong patient survival, eventually resistance will develop in nearly all patients, including the responders to the next generation hormonal therapy. The alternative splicing of AR to a constitutively active ligand-independent AR-V represents one major mechanism of resistance. Thus, identification of therapeutic agents targeting both FL-AR and AR-V may provide a novel strategy to treat ADT resistance.

Recently, much focus has been placed on the development of inhibitors that target AR NTD. Agents targeting the AR NTD are currently being explored (22–24). However, the AR NTD structure contains a high degree of intrinsic disorder and, therefore, has been difficult to target using structure-based drug design (25). AR signaling axis relies on a vast network of interacting proteins to enact their signaling effects on cells to promote growth and survival, thus taking advantage of these interacting proteins for therapeutics beyond AR itself may provide an additional avenue to combat CaP. Research into protein-protein interactions between AR and co-regulators and the development of inhibitors for these molecules will provide clinical benefit for the next-generation CaP treatment or combination therapies.

In this study, we found that inhibition of GABARAPL1 activity impairs the nuclear translocation of AR, resulting in decreased AR transcriptional output and cell proliferation of LNCaP and CWR22rv1 cells, possibly through direct scaffolding. Our data suggest that GABARAPL1 may be a new regulator of AR transcriptional activity. In line with our finding, Chen et al. reported that GABARAPL1 facilitates trafficking of Opioid receptor from ER/Golgi to plasma membranes through protein-protein interaction, which is important for trafficking of the receptor in the biosynthesis pathway (18). Constitutively active AR-V contributes to advanced CaP and therapeutic resistance. Geldanamycin, a well-established HSP90 inhibitor, has been demonstrated to effectively block ligand-dependent AR activity (26). However, it failed to modulate ARv7 protein stability or transcriptional activity (26), suggesting that AR-V may contribute to resistance to HSP90 inhibitors in clinical settings.

In summary, we provide *in vitro* and *in vivo* evidence of the potential benefit of inhibiting GABARAPL1 in FL-AR/AR-V-positive CaP cells. Although the precise mechanism for the GABARAPL1 regulation of FL-AR/AR-V transcriptional activity remains to be investigated, we have identified GABARAPL1 as a critical regulator of both FL-AR and AR-V, and GABARAPL1 may play a role in assistance in FL-AR/AR-V nuclear translocation, implying that GABARAPL1 may serve as a potential therapeutic target in FL-AR/AR-V-positive CaP.

## Data Availability Statement

All datasets generated for this study are included in the article/Supplementary Material.

## Ethics Statement

This study was performed in accordance with animal use protocols approved by the Committee for the Ethics of Animal Experiments, Shenzhen Peking University-The Hong Kong University of Science and Technology Medical Center (SPHMC) (protocol number 2011-004). All animals were handled in accordance with the guidelines of the Committee for the Ethics of Animal Experiments, SPHMC.

## Author Contributions

BS conceived of the study and participated in its design. LZ carried out the molecular and cellular studies, participated in the animal experiments. SL performed the statistical analysis. XC revised the manuscripts and the statistical analysis. WZ drafted the manuscripts. All authors read and approved the final manuscripts.

### Conflict of Interest

The authors declare that the research was conducted in the absence of any commercial or financial relationships that could be construed as a potential conflict of interest.
