# Supplementary material for: GABARAPL1 Promotes AR+ Prostate Cancer Growth by Increasing FL-AR/AR-V Transcription Activity and Nuclear Translocation
Source: Front Oncol. 2019 Nov 15;9:1254. doi: 10.3389/fonc.2019.01254 (PMC6872515; doi:10.3389/fonc.2019.01254)
Supplement: Supplementary file 1 [file Table_1.DOCX]

Supplementary Material

# Supplementary Figures and Tables

## Supplementary Figure 1

**
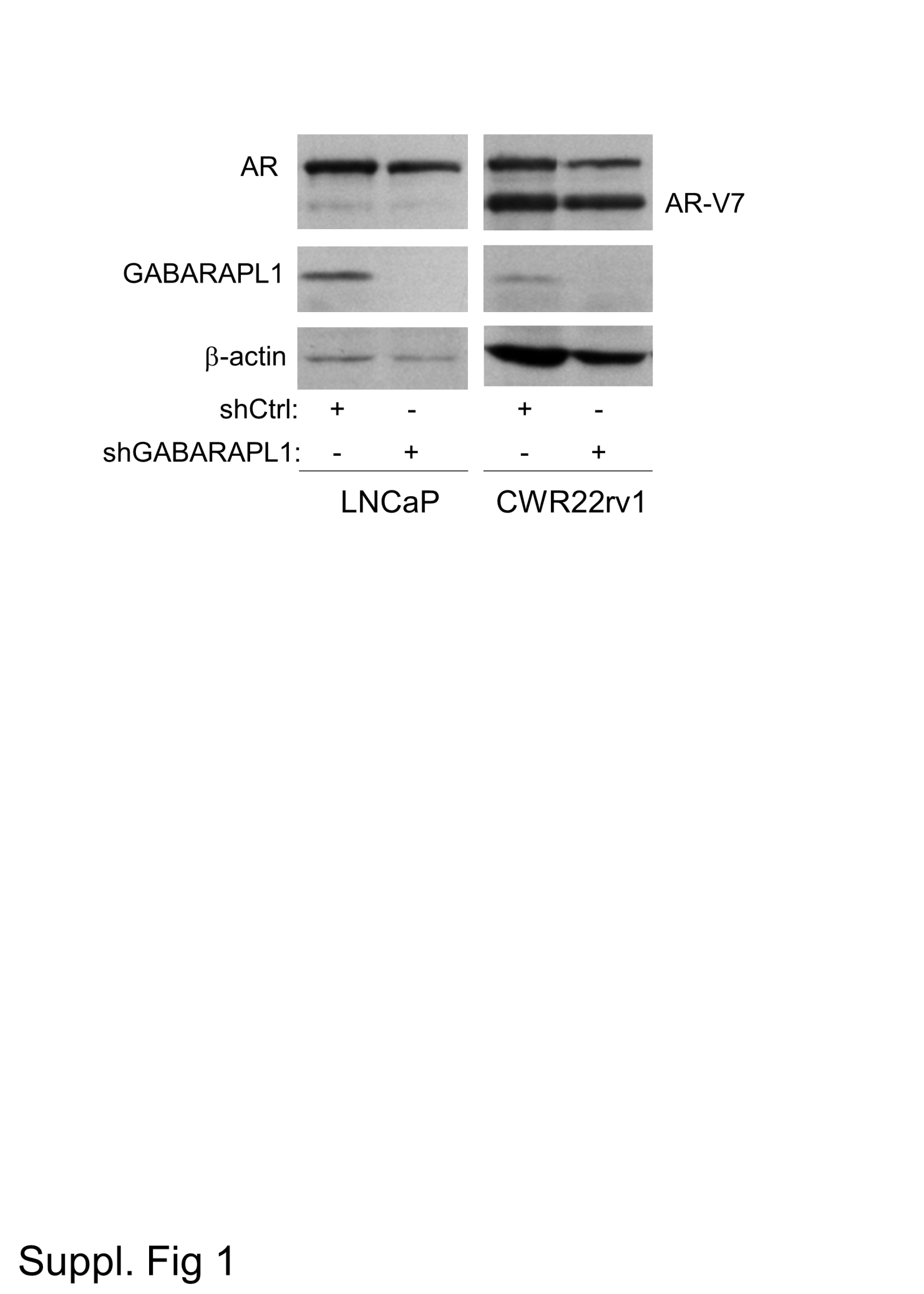
**

**Supplemental Figure 1.** Knockdown of GABARAPL1 has no effect on AR expression. LNCaP and CWR22rv1 were transduced with sh-GABARAPL1 or sh-control lentivirus, GABARAPL1 and AR expression were validated using IB analysis. β-actin was the internal control.

## Supplementary Figure 2


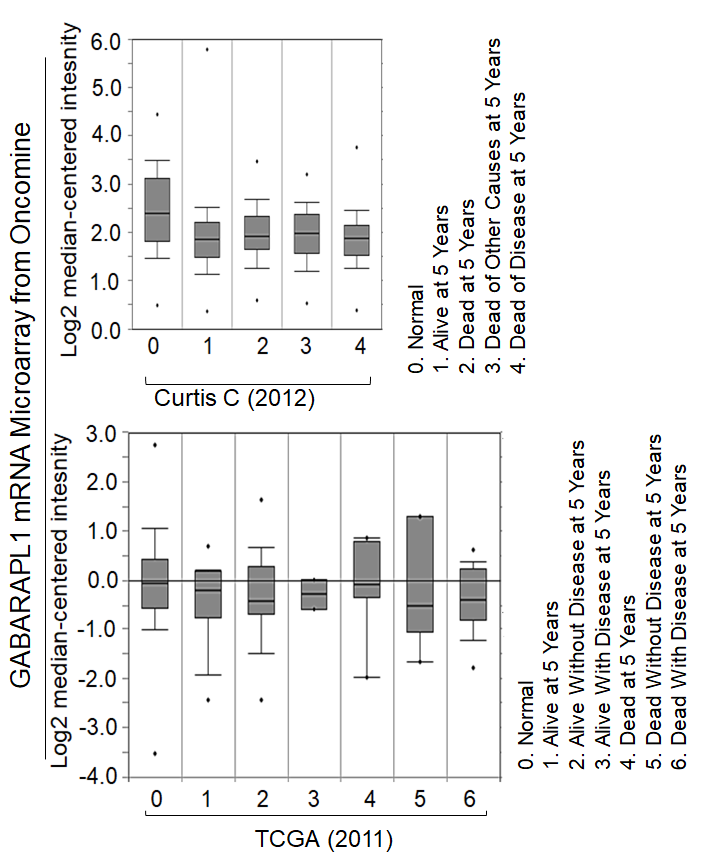


**Supplementary Figure 2.** There is no correlation between GABARAPL1 expression and 5-year survival in breast cancer cases. The data were obtained from two studies available on the Oncomine website: Curtis et al. (2012), and TCGA et al (2011).

## Supplementary Figure 3


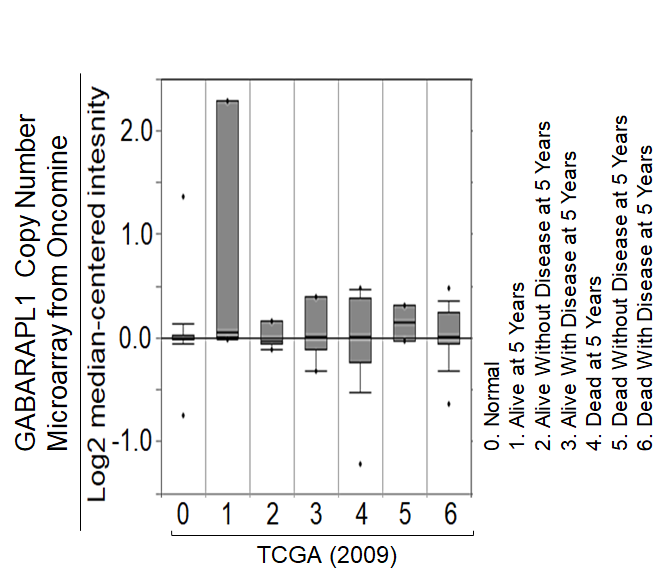


**Supplementary Figure 3.** There is no correlation between GABARAPL1 expression and 5-year survival in colon cancer cases. The data were obtained from two studies available on the Oncomine website: TCGA et al (2009).

## Supplementary Table 1. List of primers used in this study.

| **Primer Name** | **Sequence** |  |
| --- | --- | --- |
| **Primers for plasmid conduction** |  |  |
| GST-GABARAPL1 | cacgaattccatgaagttccagtacaagg |  |
|  | acagtcgactcatttcccatagacactc |  |
| p3xflag-myc-cmv-24-NTD (exon 1) | ccggaattccatggaagtgcagttag |  |
|  | atagtcgaccatgtccccgtaaggtc |  |
| p3xflag-myc-cmv-24-DBD (exon 2-3) | ccggaattcccgtttggagactgccag |  |
|  | atagtcgacggctcccagagtcatccctg |  |
| p3xflag-myc-cmv-24-LBD (exon 4-8) | attgcggccgcacggaagctgaagaaacttg | |
|  | gcgtcgacctgggtgtggaaatagatg |  |
| **Primers for qRT-PCR** |  |  |
| PSA | CCCTGAGCACCCCTATCAAC |  |
|  | TGAGTGTCTGGTGCGTTGTG |  |
| NKX3.1 | GGCCTGGGAGTCTCTTGACTCCACTAC |  |
|  | ATGTGGAGCCCAAACCACAGAAAATG |  |
| KLK4 | ATGGAAAACGAATTGTTCTGCTC |  |
|  | ATCTGGCTCCCTGGCTCTT |  |
